# Optimization of Salbutamol Sulfate Dissolution from Sustained Release Matrix Formulations Using an Artificial Neural Network

**DOI:** 10.3390/pharmaceutics2020182

**Published:** 2010-05-06

**Authors:** Faith Chaibva, Michael Burton, Roderick B. Walker

**Affiliations:** 1 Faculty of Pharmacy, Rhodes University, P.O. Box 94, Grahamstown 6140, South Africa; E-Mail: F.Chaibva@ru.ac.za (F.C.); 2 Department of Mathematics (Pure and Applied), Rhodes University, P.O. Box 94, Grahamstown 6140, South Africa; E-Mail: M.Burton@ru.ac.za (M.B.)

**Keywords:** Salbutamol sulfate, artificial neural networks, sustained release, hydrophilic matrix tablets

## Abstract

An artificial neural network was used to optimize the release of salbutamol sulfate from hydrophilic matrix formulations. Model formulations to be used for training, testing and validating the neural network were manufactured with the aid of a central composite design with varying the levels of Methocel^®^ K100M, xanthan gum, Carbopol^®^ 974P and Surelease^®^ as the input factors. *In vitro* dissolution time profiles at six different sampling times were used as target data in training the neural network for formulation optimization. A multi layer perceptron with one hidden layer was constructed using Matlab^®^, and the number of nodes in the hidden layer was optimized by trial and error to develop a model with the best predictive ability. The results revealed that a neural network with nine nodes was optimal for developing and optimizing formulations. Simulations undertaken with the training data revealed that the constructed model was useable. The optimized neural network was used for optimization of formulation with desirable release characteristics and the results indicated that there was agreement between the predicted formulation and the manufactured formulation. This work illustrates the possible utility of artificial neural networks for the optimization of pharmaceutical formulations with desirable performance characteristics.

## 1. Introduction

Pharmaceutical formulations are complex systems in which the properties and performance characteristics are influenced by numerous formulation and process factors that may not be easily understood. Pharmaceutical optimization has been defined as the implementation of systematic approaches to establish the best possible combination of materials and/or process variables under a given set of conditions that will result in the production of a quality pharmaceutical product with predetermined and specified characteristics each time it is manufactured [1]. The use of artificial intelligence such as artificial neural networks (ANN) is a rapidly growing field in knowledge discovery and data mining and has been applied in the pharmaceutical sciences for the development and optimization of dosage forms [2,3,4,5,6,7].

ANN are computational tools that emulate the interconnected neurological structures of the human brain and the ability of the human brain to learn and solve problems through pattern recognition [2]. ANN simulate the learning behavior of the human brain by modeling data and recognizing patterns for complicated multi-dimensional relationships that exist between input and output or target sets of data. Once trained an ANN can be used to predict and forecast outputs for a given a set of input conditions and may therefore be used to optimize both formulation and process variables in order to engineer and manufacture high quality, safe and effective dosage forms [8].

**Figure 1:**
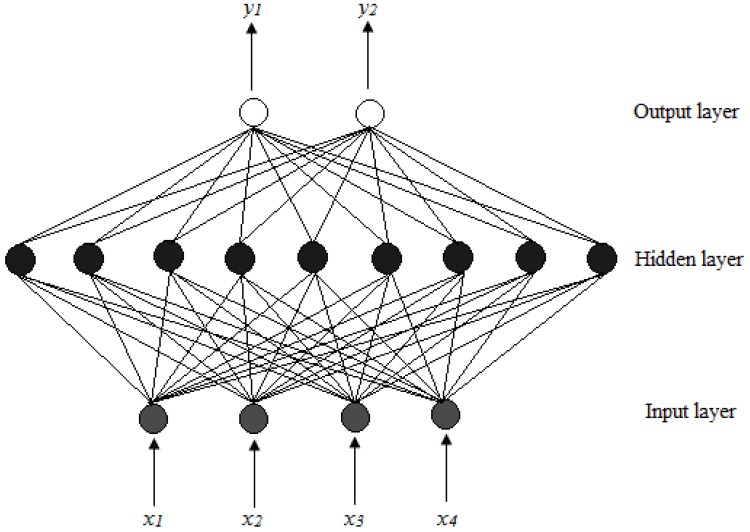
MLP architecture for a feed forward back propagation ANN where *x_1_ – x_4_* are inputs and *y_1_* and *y_2_* represent response factors [8,9].

The most commonly used network architecture for pharmaceutical applications is the multi-layer perceptron, which consists of three layers, *viz.*, an input, hidden and output layer. Each layer has a number of neurons or nodes that are fully interconnected with neurons in the neighboring layers as shown in Figure 1.

The input layer consists of one or more input nodes or processing elements that distribute input data to nodes located in the hidden layer of the ANN. Each node in the input layer can represent an independent variable, for example, an amount of polymer in a formulation or machine operating conditions such as compression force for a tablet press. The input layer does not process any information, but serves as a distribution point for information to be delivered to the hidden layer. The hidden layer can be made up of one of more layers of parallel nodes (Figure 1 shows one layer). The nodes in the hidden layer perform a weighted summation of the inputs followed by a non-linear transformation, which then relays that data to the output layer. The number of nodes in the hidden layer is critical to the efficiency of a network and if the hidden layer has too few nodes, the ANN will lack the power needed to classify the data provided to it. Conversely, if there are too many nodes, patterns in the input data will be memorized and therefore the ability of the network to interpolate data will be diminished [9,10]. The output nodes represent measureable properties of pharmaceutical formulations and may include parameters such as, for example, tablet hardness or percent drug released at different stages of a dissolution test [9,10,11]. 

The usefulness of neural networks for formulation optimization has been reported [3,4,5,6,12,13,14,15,16] and different strategies may be use used for formulation optimization. Several authors have reported the use of formulation variables such as the level of excipients used in a formulation as input or causal factors and the percent drug released at different stages of a dissolution test as response factors for ANN [5,14,15]. However, Takayama *et al.* [5] and Ibric *et al.* [16] used response factors from dissolution models to train networks and to optimize pharmaceutical formulations where it was reported that the predicted values from an ANN model were found to be in close agreement with those of experimentally generated data. Ibric *et al.* [16] used a generalized regression neural network to optimize sustained release formulation compositions for aspirin. The amount of polymer and the compression pressure were used as causal factors and the *in vitro* dissolution test sampling time points and Korsmeyer–Peppas model parameters were used as response factors. Formulation optimization using ANN models has also been performed using a generalized distance function with optimal drug release parameters being used as response factors [5]. The *f_1_* or difference and *f_2_* or similarity fit factors are typically used for the comparison of experimentally generated and predicted *in vitro* dissolution profiles when conducting optimizations with ANN models [14,16]. 

Data from experimental design methodologies are often used to construct ANN models since the experimental design approach usually ensures independence of the formulation factors used to evaluate a system [6]. The use of experimental design for developing training data for ANN has been reported and includes the use of a three factor, three level, central composite design [17], two-factor spherical second order composite experimental design [12] and a four component simplex centroid mixture design [8]. The use of empirical data from experimental design methods was therefore considered suitable for formulation optimization. 

Hydrophilic monolithic matrix devices are a popular choice for the manufacture of sustained release solid oral dosage forms due to their ease of manufacture and the extensive amount of information available regarding this well understood technology. The use of hydrophilic matrix formulations to control the release of drugs from pharmaceutical tablets is well documented [18,19,20,21]. The rate and mechanism of drug release from monolithic devices can be adjusted by the levels and types of polymer combinations that are used to manufacture a formulation. 

When hydrophilic matrix tablets are immersed in aqueous media, the polymer hydrates, swells and increases in size after which the matrix dissolves and/or erodes with time [22,23]. Early studies have shown that drug release from swellable hydrophilic matrices is dependent on the thickness of the hydrated gel layer that is formed during the swelling phase of polymer hydration [22,23]. The degree of swelling determines the diffusional path length of a drug and the thicker the gel layer the slower the rate of drug release from a matrix [24]. Drug release from hydrophilic matrix formulations occurs by drug diffusion through the gel layer and/or erosion of the tablet matrix. 

Hydroxypropyl methylcellulose (HPMC) has been used extensively for the manufacturing of tablets [25,26,27,28,29,30,31], although other matrix forming materials including Carbopol^®^ [32,33,34] and xanthan gum [18,24] have also been used. The use of Surelease^®^, an aqueous dispersion of ethylcellulose as a granulating fluid, has also been reported, where it has been shown to retard drug release from monolithic matrix formulations [35,36]. These materials were investigated as potential excipients, in combination to control the rate of salbutamol sulfate release from hydrophilic matrix tablets. 

Salbutamol sulfate, a short-acting *β_2_* agonist, is a water-soluble salt of salbutamol that is used for the treatment of bronchoconstriction and bronchospasm in patients with reversible obstructive airway disease and chronic obstructive pulmonary disease [37,38]. Salbutamol sulfate was selected as a model low-dose water soluble drug because it is stable in aqueous media and light and therefore useful for studying the impact of formulation variables on dosage performance characteristics. 

The objective of this study was to develop an optimized hydrophilic matrix formulation for salbutamol sulfate with an *in vitro* release profile that was similar to a reference formulation, *viz*., Asthalin^®^8 ER (Cipla Ltd., Mumbai, Maharashtra, India). The levels of Methocel^®^ K100M, xanthan gum, Carbopol^®^ 974P and Surelease^®^ as the granulating fluid were varied using formal experimental design, specifically a central composite design and the formulation was optimized using ANN to develop a formulation with a similar *in vitro* dissolution profile to that of the reference formulation. In addition, the ANN architecture was optimized by analyzing the efficiency of ANN that have different numbers of nodes in the hidden layer and to investigate the feasibility of using the *f_2_* similarity factor for formulation optimization. 

## 2. Experimental Section

### 2.1. Materials

Salbutamol sulfate was donated by Aspen-Pharmacare (Port Elizabeth, Eastern Cape, SA). Methocel^®^ K100M (HPMC) (Dow Chemical Company, Midland, MI, USA), Avicel^®^ PH101 (FMC BioPolymer, Philadelphia, PA, USA), xanthan gum (Aspen-Pharmacare, Port Elizabeth, Eastern Cape, SA), Surelease^®^ (Colorcon, West Point, PA, USA), Carbopol^®^ 974P NF (Lubrizol, Wickliffe, OH, USA), magnesium stearate (Aspen-Phamacare, Port Elizabeth, Eastern Cape, SA) and colloidal silica (Aspen-Pharmacare, Port Elizabeth, Eastern Cape, SA) were used as received. All other reagents were at least of analytical reagent grade and used as received.

### 2.2. Experimental design

A central composite design for formulation optimization was generated using the Model Based Calibration Toolbox of Matlab^®^ R2008a (MathWorks Inc., Natick, MA, USA) and was used for studying the impact of chosen formulation variables on the extent of drug release from matrix formulations. The independent variables, *viz.*, the amounts of Methocel^®^ K100M, xanthan gum, Carbopol^®^ 974P and percent composition of Surelease^®^ as the granulating fluid that were assessed are listed in Table 1, which shows the randomized order in which the formulations were manufactured and subsequently analyzed. *In vitro* release data from the central composite design was used to train the ANN for formulation optimization. 

**Table 1:** Formulation compositions of hydrophilic matrix tablets generated using a central composite design.

Formulation	Methocel^®^ K100M (mg)	Xanthan gum (mg)	Carbopol^®^ 974P (mg)	Surelease^®^ (% w/w)
SAL001	120	50	10	12
SAL002	60	50	10	12
SAL003	60	50	10	4
SAL004	60	50	20	12
SAL005	90	75	15	16
SAL006	60	50	10	20
SAL007	90	25	15	16
SAL008	30	75	15	16
SAL009	60	50	10	12
SAL010	90	75	5	8
SAL011	0	50	10	12
SAL012	30	25	5	16
SAL013	60	50	10	12
SAL014	30	25	15	8
SAL015	60	50	10	12
SAL016	60	100	10	12
SAL017	90	75	5	16
SAL018	30	25	15	16
SAL019	90	25	5	8
SAL020	90	75	15	8
SAL021	30	75	5	16
SAL022	30	25	5	8
SAL023	30	75	5	8
SAL024	90	25	15	8
SAL025	60	50	0	12
SAL026	90	25	5	16
SAL027	60	0	10	12
SAL028	60	50	10	12
SAL029	60	50	10	12
SAL030	30	75	15	8

### 2.3. Manufacture of sustained release matrix tablets

Surelease^®^ is a 25% w/w aqueous dispersion of ethylcellulose. Surelease^®^ was diluted for use as the granulating liquid and dispersion were prepared by accurately weighing the correct amount of dispersion on a top loading balance (Mettler Toledo Inc., Columbus, OH, USA) and diluting by weight with HPLC-grade water to concentrations of 4, 8, 12, 16 and 20% w/w. Batch sizes of 1000 tablets were manufactured for each formulation. Matrix tablets were manufactured by dry blending salbutamol sulfate and the appropriate quantities of Methocel^®^ K100M, xanthan gum, Carbopol^®^ 974P and Avicel^®^ PH101 in a Saral^®^ Rapid Mixer and Granulator (Saral Engineering Company, Mumbai, Maharashtra, India) in a 5 L bowl using a speed of 100 rpm on the main impeller for 15 min. Thereafter, 120 g of Surelease^®^ diluted to the desired concentration with water (4–20% w/w) was gradually sprayed onto the powder blend using a manual spray. The bed was agitated using speeds of 120 rpm and 1000 rpm for the main impeller and chopper, respectively. The wet mass was mixed for an additional 5 min at the same speed and removed from the granulator and allowed to dry on wax paper for 24 h at a temperature of 22 ºC. Thereafter, the granules were sieved and the fraction between 315 and 800 mm was collected and weighed, after which magnesium stearate and colloidal silica equivalent of 1% w/w and 0.5% w/w, respectively, were sieved and added to the blend that was mixed for a further 3 min at 100 rpm using the main impeller only. Finally, the lubricated granules were compressed into tablets using 9 mm biconvex punches on a Manesty^®^ B3B rotary tablet press to a uniform weight of 220 mg. 

### 2.4. In vitro dissolution studies

A VanKel^®^ Bio-Dis dissolution apparatus (VanKel Industries, Edison, NJ, USA) was used for the assessment of the *in vitro* release characteristics of salbutamol sulfate formulations. A model VK 750D digitally controlled water circulation/heater (VanKel Industries, Edison, NJ, USA) was used to maintain the temperature of the dissolution medium at 37 ± 0.5 ºC. A mesh of pore size 177 µm was used to retain the dosage form in the inner tubes and a dip speed of 10 dpm was used as the agitation rate. The dosage forms were maintained in 200 mL of buffers at pH 1.2, 4.5, 6.0 for 1, 1 and 2 h respectively and at pH 6.8 for the rest of the dissolution test and samples were collected after 1, 2, 4, 6, 8 and 12 h from commencement of the dissolution studies. 

A 2 mL aliquot of dissolution medium was removed from the dissolution vessels using an electronic pipette (Boeckel & Co (GmbH & Co), Hamburg, Hamburg, Germany) and filtered through a 0.45 µm Millipore^®^ filter (Millipore, Bedford, MA, USA). A 1.5 mL aliquot was placed in a sample vial and 100 µL of a terbutaline sulfate solution (400 µg/mL) was added to the vial such that the final concentration of the internal standard was approximately 25 µg/mL. The samples were analyzed using a validated HPLC method to determine the percent drug released at different stages of the dissolution test.

HPLC analysis was performed using a modular HPLC system that consisted of a Model P100 dual piston pump (Thermo Separation Products, San Jose, CA, USA), a Model AS100 autosampler (Thermo Separation Products, San Jose, CA, USA), which was equipped with a Rheodyne^®^ Model 7010 injector (Rheodyne, Reno, NV, USA) fitted with a 20 µL fixed volume loop and a 250 µL GASTIGHT^®^ Model 1725 syringe (Hamilton Co., Reno, NV, USA), a Linear UV/VIS-500 Model 6200-9060 detector (Linear Instrument Co., CA, USA); and a Spectra Physics SP 4600 integrator (Thermo Separation Products, San Jose, CA, USA). Separation was achieved under isocratic conditions using a mobile phase consisting of 20% v/v ACN in 18 mM phosphate buffer at pH = 4, containing 15 mM sodium octane sulphonate and a Phenomenex^®^ Hyperclone^®^ column, 5 μm, 150 mm × 4.6 mm (Phenomenex, Torrance, CA, USA) at ambient temperature (22 ºC). Samples were monitored by UV detection at 220 nm. The volume of injection was 20 μL and a flow rate of 1.0 mL/min was used for the separation.

### 2.5. Artificial neural network

Commercially available software, Matlab^®^ R2008a (MathWorks Inc., Natick, MA, USA), was used to write mathematical code for training and evaluating the ANN developed and used for formulation optimization.

A neural network composed of an input and output layer with one hidden layer, *i.e.,* a three-layer back propagation network was chosen for the purposes of this study. Four input factors corresponding to different levels of Methocel^®^ K100M (*x_1_*), xanthan gum (*x_2_*), Carbopol^®^ 974P (*x_3_*) and Surelease^®^ (*x_4_*) were used as units in the input layer of the ANN. The data generated from the central composite design was used as the training set for the ANN. The percent drug dissolved at different stages of the dissolution test (*n* = 6) was used as output the layers or the target data during ANN training. Specifically, *y_ih_* = % salbutamol sulfate released, such that *i* = 1, 2, 4, 6, 8 and 12 h. There were 180 (30 × 6) input-target data pairs that were used to train, test and validate the neural network. The data was split into three categories, *viz*., the training, test and validation data sets, where 67% of the data set was used for training and 33% as the test data. The validation set was selected from the test data and constituted 67% the test data set. 

The input and target data from *in vitro* dissolution testing was initially randomized to allow for efficient training of the network. Prior to training, the mapminmax function was used to scale the inputs and targets so that the values for these fell within a range of -1 to +1 based on the highest and lowest values in the data sets. Scaling the factors to this range is useful for efficient training of the network since it prevents bias in training if some of the values are significantly bigger or smaller than the other values in the training data set. If mapminmax is used to scale the target value, then the output of the network will be trained to produce outputs in the range -1 to +1 and these must then be converted back to original values by using the reverse of this function. 

A sigmoidal function (logsig) (Equation 1) was used as the transfer function for the hidden layer and back propagation of errors. 


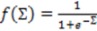
(1)

Where, Ʃ = sum of the input to the node. A purelin function was used for the output layer. The Levenberg–Marquardt algorithm for back-propagation with a gradient descent and momentum weight and bias learning function, was used to train the network. The Mean Squared Error (MSE) was used as the performance function and training was terminated after either 200 validation failures or 800 epochs or iterations of the network, whichever came first. 

Activation of the neural network was achieved by simulating the neural network using normalized training data. A post-process activation on the predicted data set was then required to convert it back to the original input range. In the final step, the predicted data was then compared with the original data set by plotting the predicted *versus* original values and computing the correlation coefficient for each of the responses in the output layer. These results were then compared, and the closer the value was to 1 then the better the predictive capability of the model. Several training sessions with different numbers of nodes (3–10) in the hidden layer were conducted in order to resolve the optimal ANN structure and each of these models were compared in this way. The optimal network architecture was then selected for further application and this final model was retrained with the entire data set to obtain a network model that could be used for formulation optimization and further simulations. 

### 2.6. Optimization procedure

Formulation optimization was conducted using the *f_2_* similarity factor (Equation 2) [39] for the comparison of dissolution profiles. 


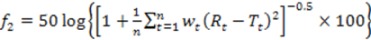
(2)

Where, *n* = is the number of dissolution sample times, *t* = the time sample index, *R_t_* = the mean percent dissolved at time *t* for the reference dissolution profile and *T_t_* = the mean percent dissolved at time *t* for the test dissolution profile. Functions for formulation optimization using simulation of the trained ANN and the *f_2_* similarity factor were written in the Matlab^®^ editor (Mathworks Inc., Natick, MA, USA). All possible permutations of the formulation variables, *viz*., Methocel^®^ K100M, xanthan gum, Carbopol^®^ 974P and Surelease^®^, within the experimental domain were generated using a brute force method and were then simulated using the trained ANN. The resultant simulated profiles were compared with the dissolution profiles of the reference formulation, Asthalin^®^8 ER (Cipla Ltd., Mumbai, Maharashtra, India). The function was used to determine the formulation that had the highest value for similarity. This formulation was subsequently manufactured and dissolution testing was conducted using USP Apparatus 3 as previously described. 

## 3. Results and Discussion

### 3.1. In vitro dissolution testing

**Figure 2:**
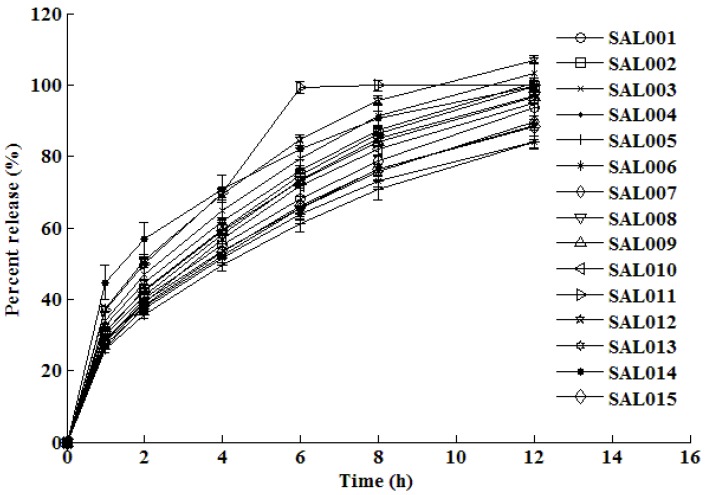
*In vitro* mean dissolution profiles of formulations SAL001 – SAL015 established from experimental design.

**Figure 3:**
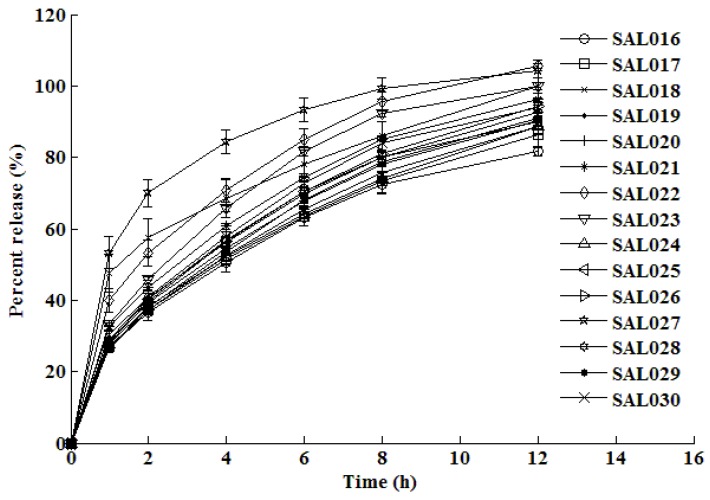
*In vitro* mean dissolution profiles of formulations SAL016 – SAL030 established from experimental design.

The dissolution test results are shown in Figure 2 and Figure 3 for the formulations manufactured using the central composite design. The dissolution profiles that were generated were subsequently used to train, test and validate the neural network. 

The *in vitro* dissolution profiles depicted in Figure 2 and Figure 3 reveal that approximately 25–47% of the dose is released within an hour of commencing dissolution testing and the remaining drug is gradually released over the next 11 h. After 12 h of dissolution testing, the percent release from all formulations ranges between approximately 83% to complete drug release. The rate of release is fairly rapid in the beginning of the dissolution test, but the rate of release decreases as the dissolution test progresses. This type of release is typical of drug release of water soluble drugs such as salbutamol sulfate, which display time-dependent release kinetics that are characterized by a diffusion controlled mechanism.

### 3.2. Training and testing ANN

The number of nodes in the hidden layer that are required to produce a good predictive network depends on the complexity of the problem to be solved, the number of nodes in both the input and output layers, and the size of the training data set. Furthermore, the amount of noise in the target data, network architecture, the required accuracy of the prediction and the training algorithm that is used are also important factors that determine the number of nodes that are required in the hidden layer [6]. The number of nodes in the hidden layer is of paramount importance when constructing an ANN model, and having too few hidden nodes decreases the learning ability of a network whereas too many hidden nodes may result in over-fitting or memorization of the training data set and a reduced ability of a network to generalize and predict accurately [6]. 

Although several approaches, including Kolmogorov’s theorem [40] and Carpenter and Hoffman’s Equation [41] have been proposed as suitable to determine the number of nodes to be included in a hidden layer of an ANN, a trial and error approach is often selected [6,42]. The trial and error approach was used to determine the optimal number of nodes for inclusion in the network, and the situation that produced the highest range of *R^2^* values for each of the response factors was selected as the optimal architecture to use. 

**Figure 4:**
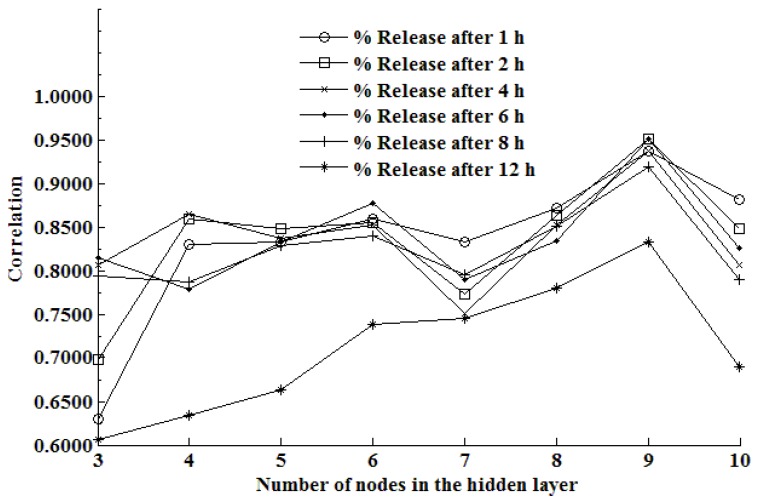
The impact of the number of nodes in the hidden layer of an ANN on the correlation coefficient at different stages of the dissolution test.

The impact of changing the number of nodes in the hidden layer of an ANN on the predictive ability of the ANN is depicted in Figure 4. 

Inspection of Figure 4 reveals that the efficiency of the network is dependent on the number of nodes in that network. It is clearly evident that the optimal number of nodes in the hidden layer for this system is nine, since this architecture produces the highest overall value of *R^2^* for all stages of the dissolution test data. As the number of nodes is increased to above nine, it is also apparent that there is a decrease in the efficiency of the network, which is likely due to overtraining of the network. 

The results reveal that when a neural network that had nine nodes in the hidden layer was used, the lowest or best validation error was observed at epoch 317 and was equivalent to 0.0249. The low error indicates that the model may be used to accurately predict the relationship between input and target data pairs and may therefore be used for prediction of outputs given a set of inputs. The correlation data for each of the different data points are summarized in Table 2.

**Table 2:** Correlation of output factors.

Output factor	*R^2^*
% Release after 1 h	0.9366
% Release after 2 h	0.9501
% Release after 4 h	0.9366
% Release after 6 h	0.9508
% Release after 8 h	0.9181
% Release after 12 h	0.8323

**Figure 5:**
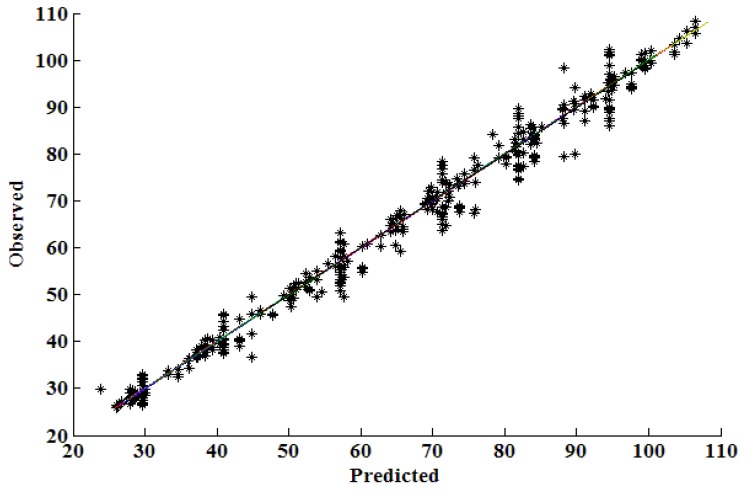
Comparison of correlation coefficients for predicted *vs.* observed data.

The results from the prediction of test data generated by the ANN *vs.* the experimentally determined or observed values in the test data set are shown in Figure 5. 

These results show that an ANN that has nine nodes in the hidden layer has good predictive capabilities and therefore may be used for formulation optimization and simulation of the impact of formulation variables on the dissolution rate of salbutamol sulfate from hydrophilic matrix formulations. The network was therefore trained with all data and was used for the formulation optimization exercise. 

### 3.3. Simulation ability of the neural network

A function for simulating the *in vitro* release profile for hypothetical formulations was written in Matlab^®^ code (Mathworks Inc., Natick, MA, USA) and used to simulate different *in vitro* release profiles. This strategy was adopted as it would be useful to determine the predictive ability of the trained network and to establish whether the network could be used to predict “unseen” data within a data set. For example, the network could be used to test a posed question such as “What would the percent drug dissolved at the different dissolution time points be from a formulation that contains 30 mg Methocel^®^ K100M, 75 mg xanthan gum, 5 mg Carbopol^®^ 974P that was granulated using a 16% w/w solution of Surelease^®^?” (provided that all the other factors and processing parameters were kept constant). The neural network is then asked to find the corresponding solution to the posed question and the percent drug released after 1, 2, 4, 6, 8 and 12 h would be 32.5%, 43.4%, 60.9%, 74.4%, 85.8% and 96.4% respectively. The corresponding *in vitro* release profile that was obtained for the manufactured formulation with the same composition revealed that 32.6%, 43.6%, 60.7%, 74.3%, 85.1% and 96.2% of salbutamol sulfate were released at the same time points. The *in vitro* dissolution profile that was generated using the simulation function was compared using the *f_2_* similarity factor, which indicated that the profiles were indeed similar because a value of 94.6 was calculated. 

### 3.4. Optimization results

The brute force method was used to predict the composition of a hydrophilic matrix formulation with the desirable release characteristics similar to the reference formulation, Asthalin^®^8 ER (Cipla Ltd., Mumbai, Maharashtra, India). A summary of the proposed formulation composition is listed in Table 3, including the predicted *in vitro* release data and the *f_2_* similarity factor for that hypothetical formulation. 

**Table 3:** Optimization formulation.

Formulation		Predicted dissolution profile	
Methocel^®^ K100M	45 mg	*y_1h_*	38.38%
Xanthan gum	30 mg	*y_2h_*	49.95%
Carbopol^®^ 974P	5 mg	*y_4h_*	65.87%
Surelease^®^	10% w/w	*y_6h_*	80.00%
Avicel^®^ PH101	105.1 mg	*y_8h_*	87.00%
Colloidal silica	0.5% w/w	*y_12h_*	95.00%
Magnesium stearate	1% w/w	*f_2_* factor	90.5

**Figure 6:**
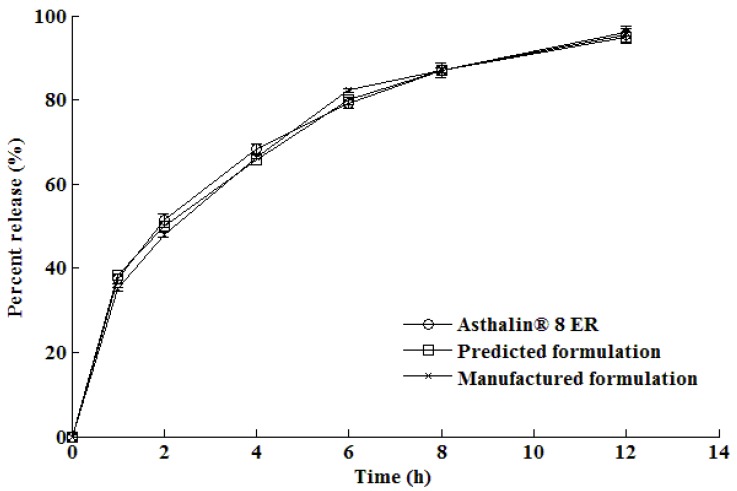
*In vitro* release profile of the optimized formulation compared with the reference formulation, Asthalin^®^ 8 ER.

The *in vitro* dissolution profile generated for the optimized formulation following manufacture is shown in comparison with the predicted and reference formulations in Figure 6. 

**Figure 7:**
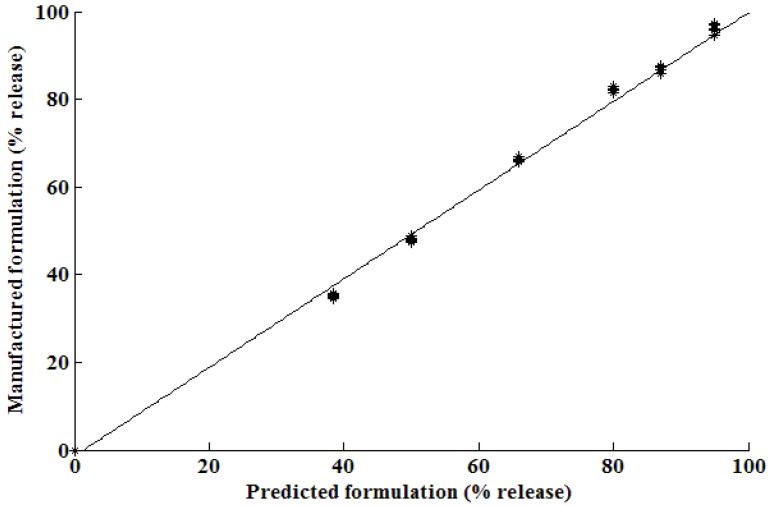
Percent drug release for the optimized *vs.* predicted formulation with y = x – 2.5 and R^2^ = 0.9964.

It is evident that there are similarities between the dissolution profiles of the manufactured formulation and that of Asthalin^®^8 ER (Cipla Ltd., Mumbai, Maharashtra, India) tablets. The *f_2_* similarity factor was calculated to be 86.0. The correlation between the manufactured formulation derived from the optimization procedure using ANN and that for the predicted formulation is shown in Figure 7. The results show that the relationship between the predicted and observed formulations was nearly linear showing excellent predictability for the optimization by use of ANN.

## 4. Summary and Conclusions

Matlab^®^ R2008a (Mathworks Inc., Natick, MA, USA) was used to write code for the training and evaluation of a neural network. Formulation variables and *in vitro* dissolution profiles from a central composite study were used for training, testing and validating the network models that were developed. 

The efficiency of the network was dependent on the number of nodes in the hidden layer and the numbers of nodes were tested by means of a trial and error approach using between three and ten neurons. The optimal number of nodes that produced a good predictive model was nine. Once the number of nodes had been established, the data was once again used to train a network that was then applied to the optimization of a sustained release matrix formulation for salbutamol sulfate. 

The *f_2_* similarity factor was used to establish whether formulation optimization had been successful by comparing ANN predicted dissolution profiles to that generated from the reference formulation, Asthalin^®^8 ER (Cipla Ltd., Mumbai, Maharashtra, India). A brute force method was applied to generate permutations for simulation into the model and the combination of formulation variables that resulted in the highest *f_2_* value was selected. The resultant model formulation was then manufactured using a wet granulation procedure. The resultant formulation was found to perform satisfactorily and an *f_2_* value of 86.0 was calculated for the comparison, clearly indicating that the dissolution profiles were similar. These results demonstrate the potential utility of ANN models for formulation development and optimization. Defining dosage form performance criteria from the outset is vitally important in developing a model formulation with the desired physical and quality characteristics. 
